# An intronic LINE-1 insertion in *MERTK* is strongly associated with retinopathy in Swedish Vallhund dogs

**DOI:** 10.1371/journal.pone.0183021

**Published:** 2017-08-16

**Authors:** Richard Everson, Louise Pettitt, Oliver P. Forman, Olivia Dower-Tylee, Bryan McLaughlin, Saija Ahonen, Maria Kaukonen, András M. Komáromy, Hannes Lohi, Cathryn S. Mellersh, Jane Sansom, Sally L. Ricketts

**Affiliations:** 1 Centre for Small Animal Studies–Ophthalmology Unit, Animal Health Trust, Kentford, Newmarket, Suffolk, United Kingdom; 2 Canine Genetics Research Group, Kennel Club Genetics Centre, Animal Health Trust, Kentford, Newmarket, Suffolk, United Kingdom; 3 Department of Veterinary Biosciences and Research Programs Unit, Molecular Neurology, University of Helsinki, Helsinki, Finland; 4 The Folkhälsan Institute of Genetics, Helsinki, Finland; 5 Department of Small Animal Clinical Sciences, College of Veterinary Medicine, Michigan State University, East Lansing, Michigan, United States of America; 6 Department of Clinical Studies, School of Veterinary Medicine, University of Pennsylvania, Philadelphia, Pennsylvania, United States of America; University of Sydney Faculty of Veterinary Science, AUSTRALIA

## Abstract

The domestic dog segregates a significant number of inherited progressive retinal diseases, several of which mirror human retinal diseases and which are collectively termed progressive retinal atrophy (PRA). In 2014, a novel form of PRA was reported in the Swedish Vallhund breed, and the disease was mapped to canine chromosome 17. The causal mutation was not identified, but expression analyses of the retinas of affected Vallhunds demonstrated a 6-fold increased expression of the *MERTK* gene compared to unaffected dogs. Using 24 retinopathy cases and 97 controls with no clinical signs of retinopathy, we replicated the chromosome 17 association in Swedish Vallhunds from the UK and aimed to elucidate the causal variant underlying this association using whole genome sequencing (WGS) of an affected dog. This revealed a 6–8 kb insertion in intron 1 of *MERTK* that was not present in WGS of 49 dogs of other breeds. Sequencing and BLASTN analysis of the inserted segment was consistent with the insertion comprising a full-length intact LINE-1 retroelement. Testing of the LINE-1 insertion for association with retinopathy in the UK set of 24 cases and 97 controls revealed a strong statistical association (P-value 6.0 x 10^−11^) that was subsequently replicated in the original Finnish study set (49 cases and 89 controls (P-value 4.3 x 10^−19^). In a pooled analysis of both studies (73 cases and 186 controls), the LINE-1 insertion was associated with a ~20-fold increased risk of retinopathy (odds ratio 23.41, 95% confidence intervals 10.99–49.86, P-value 1.3 x 10^−27^). Our study adds further support for regulatory disruption of *MERTK* in Swedish Vallhund retinopathy; however, further work is required to establish a functional overexpression model. Future work to characterise the mechanism by which this intronic mutation disrupts gene regulation will further improve the understanding of MERTK biology and its role in retinal function.

## Introduction

In the domestic dog, there are a large number of inherited and progressive retinal diseases, which are collectively termed progressive retinal atrophy (PRA). These diseases affect the photoreceptors, the retinal pigment epithelium (RPE) or both, and have been identified in many breeds [1–19]. PRA is always bilateral and almost always leads to blindness [20]. Many of these retinopathies are mirrored in human patients, and dogs have been used as animal models for human diseases such as Leber congenital amaurosis [2], retinitis pigmentosa [4] and achromatopsia [16]. Identification of each specific genetic mutation responsible provides a greater understanding of each disease and in dogs allows identification of affected individuals and carriers before breeding, which can lead to the reduction of incidence or eradication of the disease from the population. In humans this knowledge also raises the possibility of treatment of affected individuals using gene replacement therapy [21–22].

In 2014, Cooper et al. reported a novel form of PRA in the Swedish Vallhund [23]. This was the first published, peer-reviewed report of the condition, although it had been recognised by ophthalmologists in Sweden and Finland as early as the 1990s, later in the USA [24] and in the UK in 2011 (by JS (co-author)). Cooper et al. described a characteristic red-brown discolouration of the tapetal fundus, followed later by thinning of the retina and associated visual deficits. The age of onset was reported to be variable, presenting a challenge when it came to assessing the likely mode of inheritance, although it appeared most likely to be autosomal recessive. A later genome-wide association study (GWAS) investigating the genetic cause of this form of PRA identified a region on canine chromosome 17 that showed a strong reproducible association with the disease [25]. Several variants in coding regions of functional candidate genes were subsequently identified by targeted resequencing of the associated region, but none of these variants appeared to be the causal mutation. However, expression analyses of the retina in affected Vallhunds demonstrated a 6-fold increased expression of the *MERTK* gene, suggesting that this PRA in Swedish Vallhunds could be caused by a variant affecting the regulation of that gene [25].

Identification of the genetic cause of PRA in the Swedish Vallhund is particularly important because of the variation in age of onset of the condition, which makes it very challenging to reduce its incidence by selective breeding based on eye examinations alone. The aim of this study was to further investigate the genetic cause of PRA in the Swedish Vallhund, using eye examination findings in the UK and Finnish populations of Swedish Vallhunds, with the ultimate aim of providing a DNA test that can be used to identify individuals carrying a disease-associated mutation prior to breeding.

## Materials and methods

### Study samples

Samples from dogs in the study included a set of 121 Swedish Vallhunds predominantly from the UK (one dog lived in Holland, two in Canada), and a set of 145 from Finland. All samples were taken from privately owned pet dogs. Buccal mouth swabbing was conducted either by the examining veterinarian or by the owner, following informed oral and/or written owner consent. UK dogs were recruited through publicity provided by the UK Swedish Vallhund Society and interested breeders, and were examined at events held across England. The Finnish dogs were recruited for the study via word of mouth, breed club publicity, or via study-specific webpages/social media. All dogs were examined by specialist veterinary ophthalmologists (JS, AK, and Päivi Vanhapelto), or a specialist in training (RE). The examination included slit-lamp biomicroscopy (SL-15; Kowa Company Ltd, Tokyo, Japan) to examine the anterior segment and the use of topical tropicamide (Mydriacyl 1% eye drops; Alcon Laboratories UK Ltd, Camberley, UK) to achieve mydriasis and facilitate a detailed fundic examination. Funduscopy was performed using a binocular indirect ophthalmoscope (Omega 500; Heine Optotechnik, Herrsching, Germany) and a condensing lens (20D double aspheric lens; Volk Optical Inc., Mentor, Ohio, USA). Using the phenotype described by Cooper et al. (2014) [23], dogs were considered affected by the retinopathy if patches of red-brown pigment were seen starting at the periphery of the tapetal fundus as an early finding and appearing distinct from the darker pigment in the non-tapetal fundus. The presence of hyperreflective areas in the tapetal fundus was often but not always a later finding and attenuation of the retinal vasculature was only seen in very advanced cases of degeneration. The non-tapetal fundus appeared to be unaffected. The normal heterogeneity in the RPE can make the early diagnosis of this disease difficult as it appears to start at the periphery of the tapetum. In normal eyes there is considerable variation at this transitional zone with a normal variation in the degree of pigmentation in the RPE and reflectivity. To compound this, intense illumination from the ophthalmoscope can obscure the early pigmentary disturbance that occurs at this location. No specific age cut-off was used to determine that a dog was unaffected by the retinopathy, as a large variability in age at diagnosis has been reported (as early as 1.1 years and as late as 12.6 years) [23]. For the purposes of the study, a dog was considered to be unaffected if it had no signs of the retinopathy at examination, accepting that a proportion of these dogs may go on to develop the disease later. Buccal swabs were taken for DNA extraction from all dogs in the UK study set, which was carried out using a QIAamp DNA Blood Midi Kit (Qiagen, Manchester, UK). EDTA-blood samples were collected from dogs in the Finnish study set (25). The study was conducted with approval from the Animal Health Trust Ethics Committee (Project No. 33_2013) and the County Administrative Board of Southern Finland ESLH-2009-07827/Ym-23.

### Genotyping and statistical analysis

SNP BICF2G630207991 was genotyped using an allelic discrimination assay using an ABI StepOne real-time thermal cycler. Primer sequences were: CCAATCAGAAGGGAAACAGGT (forward); GCCAGGTTGAGGAATACTTGG (reverse); 5’-HEX-CTAGCTTGTGACCAGGAATTCAGGA-BHQ-3’ (C allele); 5’-FAM-AGCTTGTGATCAGGAATTCAGGAAA-BHQ-3’ (T allele). Primers used to amplify across the *MERTK* intron 1 LINE-1 insertion were CGTTTACCTAATTACAGTCCCAGA (forward primer) and CAACTTAATGTGTGCTGCTTAGGA (reverse primer). For the PCR reaction we used PrimerSTAR GXL DNA Polymerase (Takara Clontech, Saint-Germain-en-Laye, France) that is optimised for difficult and large amplicons. PCR comprised 30 cycles of 98°C for 10 seconds, 60°C for 15 seconds, and 68°C for 8 minutes. The *MERTK* intron 1 LINE-1 insertion was genotyped by fragment analysis on an ABI 3130XL Genetic Analyzer using HOT STAR Taq (Qiagen, Manchester, UK); a common forward primer (GAATAAACACATCCCTGGCATT); a wild-type reverse primer (TGCAACTTAATGTGTGCTGCT); a mutant reverse primer (CTACTCTTCCGCCATCTTGCT); and the addition of 0.05 μM (final concentration) fluorescently labelled forward primer. PCR cycling consisted of an initial 95°C denaturation step for 5 minutes, followed by 35 cycles of 95°C for 30 seconds, 60°C for 30 seconds, and 72°C for 1 minute. Final elongation was 72°C for 5 minutes. The association with retinopathy was tested using the Fisher’s exact test. Linkage disequilibrium LD among variants was assessed using pairwise correlation (r^2^). We used logistic regression and log-likelihood ratio tests to compare a general model with a linear per allele model to assess the shape of the association between the LINE-1 insertion and retinopathy and to compute the odds ratio for the pooled UK and Finnish study sets. All of the above statistical analyses were conducted using STATA 10.0 (College Station, TX, USA).

### Whole genome sequencing (WGS) and analysis

WGS was conducted for one UK case and one Finnish case and its affected sire. Since the UK DNA sample was derived from a buccal swab, it was checked for integrity (percentage of dog DNA) by obtaining a random distribution of WGS reads (~10,000) using a MiSeq platform (Illumina). The UK case selected for WGS showed an aligned read percentage of 89.4%. (As samples from the Finnish dogs were derived from whole blood, this stage could be omitted.) PCR-free library preparation and paired-end sequencing (100 bp reads) was carried out for the UK case at the High-Throughput Genomics Group, Wellcome Trust Centre for Human Genetics, University of Oxford, UK on one lane of an Illumina HiSeq4000. The Finnish dogs were sequenced at the University of Bern, Switzerland using a PCR-free library preparation and pooled in a set of eight dogs sequenced over six lanes of a HiSeq2500. Sequence reads were aligned to the canine reference genome (CanFam 3.1) using BWA-MEM [26] and SNP/insertion-deletion (in-del) calls were made by GATK HaplotypeCaller (v.3.4) [27] using GATK best practices. WGS data can be found in the European Nucleotide Archive, study accession numbers PRJEB21485 and PRJNA394814.

### Characterisation of LINE-1 insertion

A library was constructed from multiple PCR amplicons of a single case for the LINE-1 insertion using the above flanking primers and a modified version of the Illumina Nextera XT kit. After the initial tagmentation stage, a 1X AMPure XP bead purification was performed (Beckman Coulter, High Wycombe, UK), and in the following PCR stage KAPA HiFi HotStart ReadyMix (Kapa Biosystems, Boston, MA, USA) was used instead of the Nextera PCR Master Mix (NPM). Cycling conditions were 72°C for 3 minutes, 98°C for 30 seconds, then 12 cycles of 98°C for 10 seconds, 63°C for 30 seconds, and 72°C for 3 minutes, with a final 72°C for 1 minute followed by a hold at 10°C. After the final bead purification the product was resuspended in 25 μl ultrapure water. The library was quantified by qPCR using the KAPA library quantification kit (Kapa Biosystems, Boston, MA, USA). Sequencing of the library was performed on one run of an Illumina MiSeq instrument, generating paired-end reads of 151 bp in length. *De novo* sequence assembly was carried out using SOAPdenovo2 [28] to create contigs from a subset of the MiSeq data.

## Results

We first sought to replicate the previously published association for retinopathy in the Swedish Vallhund on canine chromosome 17 by genotyping the top SNP from the previously published GWAS (BICF2G630207991 at CanFam 3.1 co-ordinate 36,400,229) [25] in a UK-based set of 24 retinopathy cases and 97 controls. This SNP showed a strong statistical association with retinopathy in the UK dogs—P-value for Fisher’s exact test: 4.7 x 10^−9^—and was directionally consistent with Ahonen et al. [25]. Genotype distributions for SNP BICF2G630207991 in the case and control groups of the UK study set are shown in Table 1A.

**Table 1:** Genotypes for top GWAS SNP and LINE-1 insertion in Swedish Vallhunds from the UK and Finland.

**Table 1a UK dogs (24 cases and 97 controls)**				
	**Retinopathy status**			
**SNP_BICF2G630207991 genotype^a^**	**Case n (freq.)**	**Control n (freq.)**	**Total**	
T/T	3 (0.13)	25 (0.26)	28	
T/C	6 (0.25)	67 (0.69)	73	
C/C	15 (0.63)	5 (0.05)	20	P-value: 4.7 x 10^−9^
**LINE-1 insertion genotype**				
Wildtype	3 (0.13)	28 (0.29)	31	
Heterozygote	6 (0.25)	67 (0.69)	73	
LINE-1 ins	15 (0.63)	2 (0.02)	17	P-value 6.0 x 10^−11^
**Table 1b Finnish dogs (49 cases and 89 controls)**				
	**Retinopathy status**			
**SNP_BICF2G630207991 genotype^a^**	**Case n (freq.)**	**Control n (freq.)**	**Total**	
T/T	0 (0.00)	26 (0.29)	26	
T/C	7 (0.14)	51 (0.57)	58	
C/C	42 (0.86)	12 (0.13)	54	P-value 1.3 x 10^−17^
**LINE-1 insertion genotype**				
Wildtype	0 (0.00)	28 (0.31)	28	
Heterozygote	7 (0.14)	51 (0.57)	58	
LINE-1 ins	42 (0.86)	10 (0.11)	52	P-value 4.3 x 10^−19^

^**a**^ For SNP_BICF2G630207991 the risk allele is C

**Fig 1:**
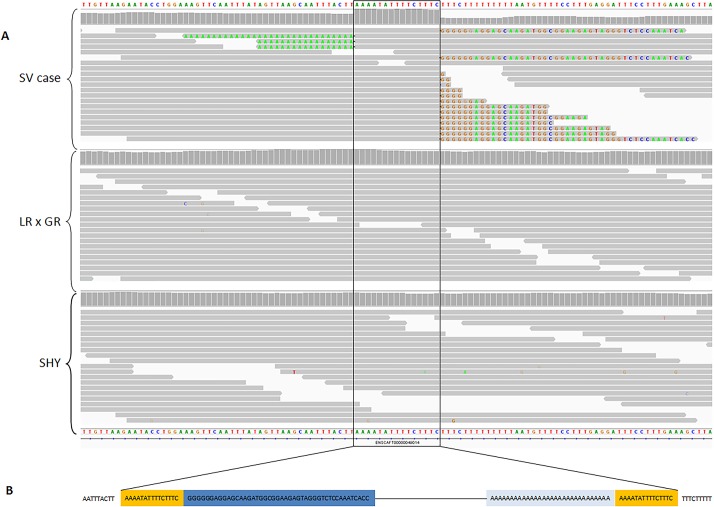
IGV Illustration of the LINE-1 insertion in intron 1 of the *MERTK* gene. **A**. The upper panel shows the Swedish Vallhund case, and the middle and lower panels represent two control WGS for comparison (a Labrador-Golden retriever cross and a Siberian Husky, respectively). The 15 bp duplicated sequence that is the LINE-1 insertion point is boxed. The CanFam 3.1 reference sequence (Boxer) is shown above and below the figure. **B**. Diagram to clarify the LINE-1 insertion in the Swedish Vallhund case. The 15 bp duplication is shown in orange, and soft-clipped bases in sequence reads at the start and end of the LINE-1 insertion (from the upper panel of **A**) are shown in dark and light blue, respectively. The line joining these two sequences represents the 6.401 kb of LINE-1 insertion sequence.

Next we utilised PCR-free WGS to generate complete sequence spanning the genomic interval defined by the previous GWAS data [25] (CanFam 3.1 chr17:34,421,056–40,576,539) using a UK case that possessed two copies of the risk allele (C) at SNP BICF2G630207991. Median read depth of the UK case was 21X with 97% of bases achieving at least 10X read depth; 92.2% of reads mapped to the reference genome. Median read depth of the Finnish dogs were 24X for the case and 21X for the affected sire with 97% of bases achieving at least 10X read depth; 99% of the reads mapped to the reference genome. Given previous evidence for involvement of the *MERTK* gene, we initially focused on the region directly encompassing *MERTK* (chr17:36,336,729–36,445,380) by visually scanning sequence reads in IGV (Integrative Genomics Viewer) [29]. We initially compared the Swedish Vallhund case with WGS from three dogs each of a different breed that have been generated for the study of other diseases in our laboratory and were not expected to share the retinopathy mutation. This analysis revealed a 15 bp region of increased read depth followed by a sharp truncation of reads in intron 1 of the *MERTK* gene (chr17:36,338,043–36,338,057; 62,172 bp upstream of SNP BICF2G630207991) and 1,006 bp downstream of the end of the first predicted exon, suggestive of an insertion, or duplication (Fig 1). This feature was present in the retinopathy case but was not present in 49 additional WGS of different breeds. The same insertion was also found in the WGS from the Finnish Swedish Vallhunds. Both the Finnish case and its affected sire were homozygous.

**Fig 2:**
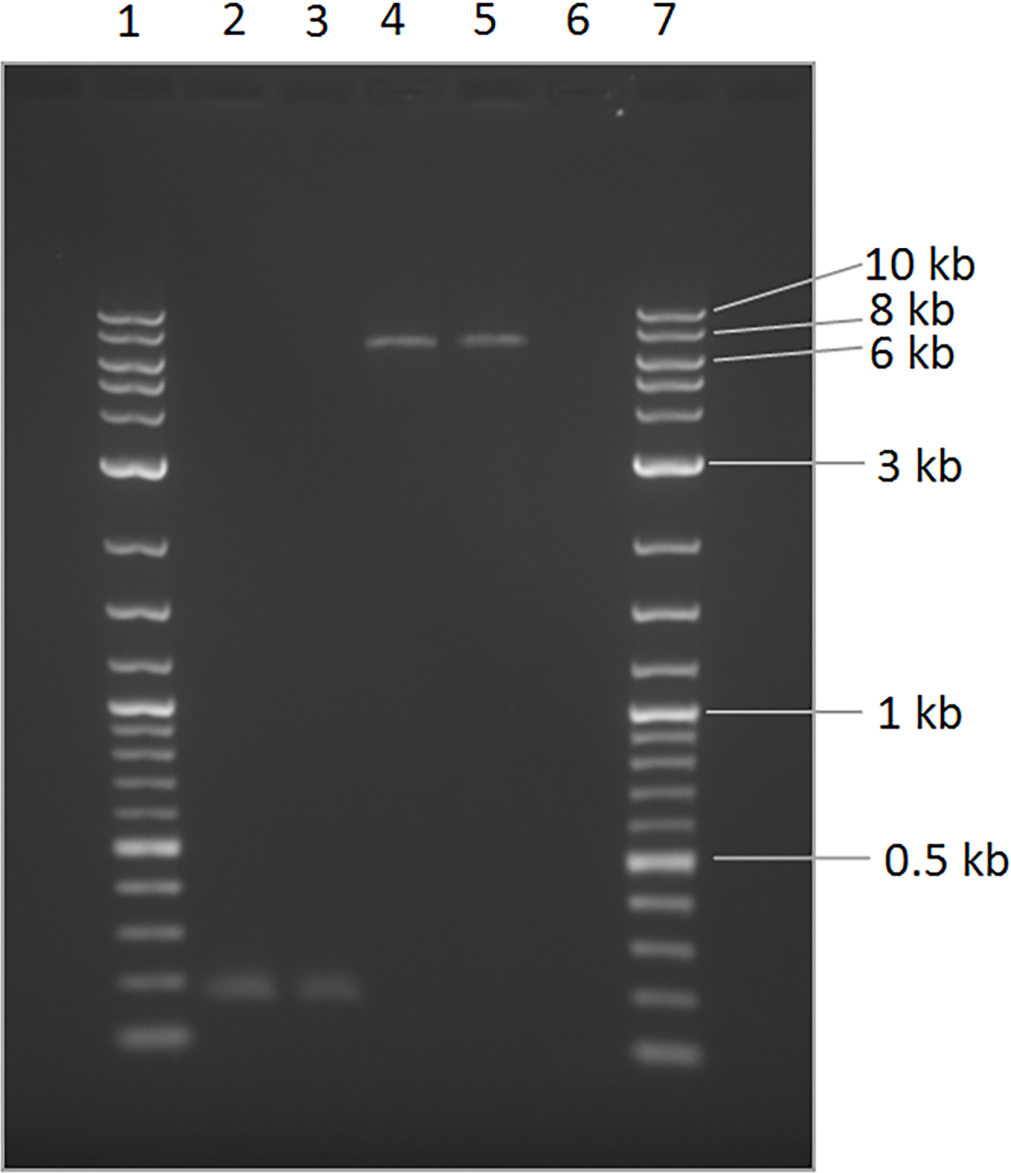
Agarose gel electrophoresis analysis of PCR amplification of the LINE-1 insertion in UK Swedish Vallhund retinopathy cases and controls. Lanes 1 and 7 are a 2-Log DNA ladder (New England Biolabs, Hitchin, UK); Lanes 2 and 3 are controls negative for the LINE-1 insertion; Lanes 4 and 5 are cases positive for the insertion; Lane 6 is a non-template control.

BLASTN analysis [30] of the duplicated segment and part of the sequencing reads that were soft-clipped by BWA (Fig 1) revealed multiple hits across the canine genome with a LINE-1 element as the top signal, indicating that the insertion involves a retrotransposon. To further characterise the insertion, we amplified across it using flanking primers. Agarose gel analysis of a subset of two cases and two controls revealed an approximate 6–8 kb fragment for cases, and the 180 bp CanFam 3.1 reference-predicted fragment size for controls (Fig 2). We attempted to sequence the 6–8 kb case amplicon using a MiSeq instrument (Illumina) and used *de novo* assembly to construct a 6.401 kb contig of the insertion between the two duplicated segments, ending with a low complexity run of A nucleotides that we were unable to fully decipher. BLAST analysis of this contig aligns with 99% identity to the canine L1-Y_CF consensus sequence found in RepBase [31–32]. Analysis of the insertion sequence using NCBI ORF Finder revealed a full-length LINE-1 element comprising the 15 bp target site duplication (Fig 1), a 5’UTR, 900 bp 5’ ORF1, a 49 bp intergenic spacer, a 3,828 bp 3’ ORF2, a 3’UTR and poly(A) tail. The LINE-1 element has been inserted in the same orientation as the *MERTK* gene.

We developed a genotyping assay for the LINE-1 insertion and tested it for association with retinopathy in the UK set of 24 cases and 97 controls and found a strong statistical association (P-value 6.0 x 10^−11^) (Table 1A). The LINE-1 insertion was correlated to the top GWAS SNP at an r^2^ of 0.94 in the UK study set. Log likelihood ratio tests based on logistic regression suggested that the association was non-linear, indicating an autosomal recessive mode of inheritance that supports previous suggestions [23]. We consequently sought to replicate the LINE-1 insertion association in the Finnish samples that had been part of the original GWAS study [25]. In a set of 49 cases and 89 controls, the LINE-1 insertion showed a strong association with retinopathy (P-value 4.3 x 10^−19^) and this association was two orders of magnitude stronger than the top GWAS SNP in this study set (P-value 1.3 x 10^−17^) (Table 1B). The correlation between the LINE-1 insertion and top GWAS SNP was r^2^ of 0.97 in the Finnish study set and, as for the UK study set, the association appeared non-linear. In a pooled analysis of both study sets (comprising a total of 73 cases and 186 controls) using logistic regression with adjustment for study, the LINE-1 insertion was associated with a ~20-fold increased risk of retinopathy (odds ratio 23.41, 95% confidence intervals 10.99–49.86, P-value 1.3 x 10^−27^).

We also screened the remainder of the genomic region spanning the *MERTK* gene (chr17:36,336,729–36,445,380) and found two additional intronic variants that were present in only the retinopathy case compared to the 49 other WGS. One has been previously tested by Ahonen et al. [25] (CanFam 3.1 co-ordinate chr17:36,410,244) and was found to be weakly associated with retinopathy, and subsequent genotyping of the other (CanFam 3.1 co-ordinate chr17:36,399,739) in the UK study set of 24 cases and 97 controls revealed a strong statistical association equal to SNP BICF2G630207991 (P-value 4.7 x 10^−9^) to which it showed perfect correlation. However, as the association was weaker than for the LINE-1 insertion this variant was not considered further. Testing of the LINE-1 insertion in a multi-breed panel of 92 dogs comprised of 31 breeds found no evidence of the insertion in these breeds. We also compared the Vallhund WGS to the 49 WGS of other breeds for the whole associated interval above. This identified 2,108 variants, of which 108 were homozygous for one allele in the Vallhund and homozygous for an alternative allele in the dog reference genome and all other 49 WGS. Two of these were coding indel variants in the following genes: *FOXI3* (chr17:38,032,793) and *PROM2* (chr17:34,827,666), but neither are plausible candidates for retinopathy. The *FOXI3* variant comprised a 9 bp insertion located within a tandem repeat sequence not well conserved following CLUSTAL [33] alignment of the canine protein sequence with that of 27 placental mammals (data not shown). The *PROM2* variant was a 25 bp insertion also situated within a tandemly repeating sequence that was polymorphic amongst the 49 WGS and was not well conserved following CLUSTAL alignment of the canine protein sequence with 31 placental mammals (data not shown).

## Discussion

Our study has independently replicated a previously published association with retinopathy and the *MERTK* locus in the Swedish Vallhund and has identified a LINE-1 repeat element insertion in intron 1 of the *MERTK* gene that shows a strong statistical association with retinopathy. Whilst the LINE-1 insertion is a very strong candidate causal mutation, the mechanism underlying the association requires further elucidation, as we did not have access to tissue samples from affected UK dogs with which to conduct RNA or protein analyses. However, our findings are consistent with observations from the previous genetic study, where interestingly, whilst there was no structural difference in *MERTK* mRNA transcripts amongst two Swedish Vallhund retinopathy cases from Finland (neuro-retina and RPE) and an unaffected dog of a different breed, an analysis of *MERTK* expression levels demonstrated a 6.5-fold increased level of *MERTK* expression in four Vallhund cases when compared to two controls of other breeds [25].

LINE-1 retrotransposons are common in mammalian genomes [34], including humans and dogs [35,36] and their integration into intronic genomic DNA has been associated with several diverse diseases in both species, although mostly with a concurrent alteration in structure of the mRNA of the gene affected and/or with a significant decrease in gene expression [35, 37–43]. The full-length LINE-1 insertion shown in this study is similar to one associated with reduced expression of the aryl hydrocarbon receptor gene involved in liver metabolism in Irish Wolfhounds, although this LINE-1 sequence contains frame shifts in both ORFs [42], whereas the LINE-1 insertion identified in the Vallhund appears intact. There are several potential mechanisms by which the insertion of a LINE-1 element can affect expression of nearby genes; by introducing regulatory elements or promoters that could act as enhancers, or by the creation of 5’ and 3’ truncated mRNAs as well as the intact mRNA that could affect/mask the assessment of normal gene expression [37, 38, 44]. It could also be possible that the LINE-1 insertion disrupts a cis-acting regulatory element within intron 1 of the gene. To help elucidate this, functional experiments using genome editing techniques such as CRISPR could potentially examine *MERTK* expression levels in cell lines established from retinal tissue of affected dogs with subsequent partial and full removal of the LINE-1 insertion. Further characterisation of *MERTK* mRNA isoforms in affected Swedish Vallhunds will also be important to confirm that the increased expression of *MERTK* shown in the previous study [25] is that of intact *MERTK* and not alternative truncated transcripts that could affect the normal function of the MERTK protein. Protein expression studies would also be useful to establish whether MERTK levels are different between affected and unaffected Swedish Vallhunds.

The role of MERTK in driving the epithelial cell phagocytosis of apoptotic cells (efferocytosis) of photoreceptor outer segments by the RPE as part of the regular shedding and replacement of these cells in the retina is well established [45] and was first confirmed in the Royal College of Surgeons (RCS) naturally occurring rat model of retinal dystrophy. A mutation in the *MERTK* gene was identified as causing a deficiency in this process, leading to the build-up of cellular debris and the deterioration of the photoreceptors [46]. Following this, and the identification of mutations in humans with retinitis pigmentosa where loss of MERTK function has also been demonstrated [47, 48], its biology has been widely investigated due to the potential of gene therapy to correct deficiencies in *MERTK* expression caused by mutations in the gene [49]. However, the scenario that is suggested by the combined results of this and the previous study is converse to the findings in human and rat and involves a similar disease phenotype caused by suggested upregulation of the *MERTK* gene. The possible mechanisms for how this could occur have been discussed in the previous study [25]. MERTK is a transmembrane protein of the MER/AXL/TYRO3 receptor tyrosine kinase family, and is also a proto-oncogene that appears upregulated in cancers such as mammary tumours, cell lines from which have been shown to demonstrate an enhanced level of efferocytosis [50]. In the retina, MERTK has two forms; a membrane-bound form that initiates efferocytosis and a soluble form (sMERTK) that inhibits this process in a negative feedback loop, ensuring that phagocytosis in the RPE occurs only over a limited time duration [51, 52]. Therefore efferocytosis of photoreceptor outer segments is closely controlled in RPE cells, but how any overexpression of *MERTK* would have an effect on this delicate balance between the MERTK and sMERTK molecules needs to be clarified. Indeed the establishment that it is intact *MERTK* that is being overexpressed in affected Vallhunds is important, as is whether this occurs before the development of disease and that it is therefore a cause and not a consequence of retinopathy. Despite these uncertainties, the identification of the genetic defect underlying this naturally occurring canine overexpression model for *MERTK* is a novel and exciting finding and could therefore inform future studies of *MERTK* in both human and canine retinal disease.

In both study sets combined, 6% of controls and 22% of cases were discordant in terms of their genotype-phenotype status and therefore warrant further discussion. In the UK study set these are two controls that genotyped homozygous for the LINE-1 insertion and were free of ocular disease at entry into the study in autumn 2014 (Table 1A). At this time one of the dogs was 3 years 8 months old and the other 7 years 11 months old. The younger dog was re-examined in November 2015 by JS (co-author) at 4 years 11 months old and was still found to be free of ocular disease. In the Finnish study set there were 10 controls that were homozygous for the LINE-1 insertion and were free of ocular disease at examination (Table 1B). The age of last eye examination for these 10 dogs ranged from 4.4–12.7 years (median 5.75) and so it is again possible that clinical signs are yet to appear. This is consistent with the variability in age of onset reported by Cooper et al. [23], who reported changes as early as 1.1 years and as late as 12.6 years of age. Cooper et al. suggest that possible explanations for this large variation in age of onset include both genetic and environmental modifiers, and that these may also be responsible for the variations in phenotype. Another possible explanation for the variation in phenotype could lie in the mosaicism of the RPE, which has marked variation between cells in the content of melanin and lipofuscin. This is thought to arise by normal mechanisms regulating gene expression, and could certainly account for some of the differences in phenotype [53]. The heterogeneous phenotype in the affected Vallhunds could be due to the unusual overexpression of *MERTK*. Interestingly, a recent study has identified the *Tyro3* gene as a genetic modifier of retinopathy in the *Mertk* knockout mouse, with allelic variation at *Tyro3* moderating the clinical manifestation of the disease [54]. There are also suggestions that the activity of transposable elements varies within tissues, so any mechanism by which a gene-specific LINE-1 insertion exerts effects on nearby gene expression may be variable at a cellular level and modulated by epigenetic means [55].

By contrast, three of the UK cases do not carry the LINE-1 insertion and six are only carriers (Table 1A); amongst the Finnish cases there are seven carriers of the LINE-1 insertion. We did not find any statistical evidence in our study sets that carriers have an increased risk of disease and our analyses strongly signified an autosomal recessive mode of inheritance. Overall our data showed 83% concordance for cases and 94% for controls, with 57 out of the 69 dogs that possessed two copies of the LINE-1 insertion being affected by retinopathy (Table 1). This level of concordance is broadly similar to that found for other forms of PRA where the discordancy in cases is suggested to be due to genetic heterogeneity of the condition [9–11, 56]. Genetic heterogeneity is a scenario typical to PRA in many breeds [1] and indeed in human retinitis pigmentosa [57] and so may also be the situation for retinopathy in the Vallhund. This condition does represent a form of PRA involving both the RPE and the photoreceptors, although its clinical presentation and the involvement of MERTK suggests that this retinopathy is primarily related to a defect in the RPE. Future studies involving the discordant cases in this study may uncover additional regions of the canine genome that are involved in the disease in the Swedish Vallhund.

## Conclusions

We have identified a LINE-1 insertion in intron 1 of the *MERTK* gene that is strongly associated with retinopathy in the Swedish Vallhund, and which is a strong candidate for a causative mutation. This study adds further support for regulatory disruption of the *MERTK* gene in Swedish Vallhund retinopathy, although to establish this functional overexpression model more work is required to further investigate MERTK biology in retinal function. Further characterisation of how this LINE-1 insertion modulates *MERTK* expression may improve the understanding of its regulation and whether there are motifs disrupted by its insertion. Our results will enable the development of a DNA-based breeding tool that directly assays this mutation in the Swedish Vallhund. This will help breeders to avoid producing clinically affected dogs and will also aid veterinary ophthalmologists with differential diagnosis of the condition in the breed.
